# An Ensemble Method for Missing Data of Environmental Sensor Considering Univariate and Multivariate Characteristics

**DOI:** 10.3390/s21227595

**Published:** 2021-11-16

**Authors:** Chanyoung Choi, Haewoong Jung, Jaehyuk Cho

**Affiliations:** 1 School of Statistics and Actuarial Science, Soongsil University, Seoul 06978, Korea; cksdud150@soongsil.ac.kr; 2 School of Electronic Engineering, Soongsil University, Seoul 06978, Korea; mbmb7777@soongsil.ac.kr

**Keywords:** missing data, environmental sensor, univariate and multivariate imputation, machine learning, ensemble method

## Abstract

With rapid urbanization, awareness of environmental pollution is growing rapidly and, accordingly, interest in environmental sensors that measure atmospheric and indoor air quality is increasing. Since these IoT-based environmental sensors are sensitive and value reliability, it is essential to deal with missing values, which are one of the causes of reliability problems. Characteristics that can be used to impute missing values in environmental sensors are the time dependency of single variables and the correlation between multivariate variables. However, in the existing method of imputing missing values, only one characteristic has been used and there has been no case where both characteristics were used. In this work, we introduced a new ensemble imputation method reflecting this. First, the cases in which missing values occur frequently were divided into four cases and were generated into the experimental data: communication error (aperiodic, periodic), sensor error (rapid change, measurement range). To compare the existing method with the proposed method, five methods of univariate imputation and five methods of multivariate imputation—both of which are widely used—were used as a single model to predict missing values for the four cases. The values predicted by a single model were applied to the ensemble method. Among the ensemble methods, the weighted average and stacking methods were used to derive the final predicted values and replace the missing values. Finally, the predicted values, substituted with the original data, were evaluated by a comparison between the mean absolute error (MAE) and the root mean square error (RMSE). The proposed ensemble method generally performed better than the single method. In addition, this method simultaneously considers the correlation between variables and time dependence, which are characteristics that must be considered in the environmental sensor. As a result, our proposed ensemble technique can contribute to the replacement of the missing values generated by environmental sensors, which can help to increase the reliability of environmental sensor data.

## 1. Introduction

The concept of a smart city has become a trend, with rapid urbanization occurring worldwide. Accordingly, various technologies that are necessary for smart cities, such as the Internet of Things, machine learning, and big data applications have been developed. Among the various smart city technologies, interest in the deployment of applications for environmental pollution monitoring is increasing [1,2]. In addition, the environment is deteriorating due to economic activity, rapid urbanization, and increased energy consumption [3]. The World Health Organization (WHO) announced that air pollution, soil quality, and water quality are the biggest environmental risk factors for health. Air pollutants that penetrate through the respiratory tract and blood vessels adversely affect the lungs, heart, and brain [4,5]. As a result, people are increasingly interested in how the environment affects their health. Accordingly, interest in and demand for environmental sensors that are capable of measuring air pollutants are increasing [6]. The reliability of environmental sensors hinders the achievement of accurate measurements of environmental pollution. Since one of the factors impairing this reliability is the occurrence of missing values, the importance of the process of handling missing values has recently been highlighted [7]. Missing values mean that there is no data or that data is incomplete [8]. Missing values can be caused by various reasons, such as unstable communication from the sensor and errors in the sensor device (measurement range, rapid change in data), etc. [9,10]. Internet of Things (IoT)-based environmental sensors always have a problem with data omission, due to the inevitable instability of communication, which is due to dynamically changing communication methods. With these data, biased parameter estimation in analysis and predictive models can cause problems, due to low analysis quality and accuracy [11]. Therefore, to increase reliability in IoT-based environmental sensors, it is necessary to consider a method for processing missing values.

When a missing value occurs in the environmental sensor, there are two main ways of dealing with it: deletion and imputation. First, deletion is literally a method of omitting missing values. This procedure is usually only justified when large amounts of data are available. In general, 5% or less of the total data are within the range that can be deleted [12]. In contrast, imputation is a method of replacing missing values with estimates [13]. Based on the estimation method, various substitution methods exist, such as mean substitution, regression, and last observation carried forward (LOCF) [14,15]. Since deletion has the potential to cause losses in the final result, deletion is not suitable as a method of processing missing values of environmental sensors, which require an improvement in data reliability [16]. Therefore, it is necessary to establish a new and systematic process, related to imputation, that is suitable for environmental sensors, which require a sensitive and real-time performance.

To find an imputation method that is suitable for environmental sensors, 22 environmental sensor devices were fabricated. These devices include a sensor that can measure 10 types of environmental substance, such as CO, CO_2_, PM2.5, PM10, TVOC, H_2_S, NO_2_, and NH_3_, as well as temperature and humidity. These devices were separately installed in two buildings within the university. Since a given indoor environment can change depending on the measurement location, devices were installed separately to check whether the sensor showed good linearity in different environments. Two devices showing linearity were selected, and the experiment was conducted. The environment was set with one device for reference and the other device for directly generating missing values.

The measured time series data of the environmental sensors had a continuous characteristic and were sequentially collected [17]. These successive observations had an autocorrelation with each other. In addition to this, the manufactured environmental sensor measured various environmental substances at the same time in the form of an integrated device, and the correlation between specific environmental substances could be checked according to changes in the environment. 

When imputing missing values in environmental sensor data, two methods can be used, as follows: a method using time dependence and a method using correlations between variables. The method that considers the time characteristics of univariate input is called univariate time series imputation, and the method of imputation that considers the dependency between other variables, when two or more are measured, is called multivariate imputation [18].

In existing papers, the multivariate imputation method has been more frequently used than the univariate imputation method (which has rarely been used), when dealing with multivariate data [19,20,21]. However, since the time series characteristics of each variable can be extracted from multivariate data, this paper attempted to use univariate imputation and multivariate imputation simultaneously, in one piece of multivariate data. This considered the environmental sensor characteristics and the correlation between time series observations, as well as the dependency between variables. Since univariate and multivariate characteristics are different for each datum, depending on the situation, it may be advantageous to use only one of the two methods, or it may be more appropriate to substitute one, considering both methods at the same time. Therefore, the authors attempted to create various cases in which missing values can occur, and suggest which technique is appropriate for each case.

Therefore, we divided the cases where missing values occur into several categories. We have measured these using environmental sensors since 2020 and identified the types of missing values that occur frequently, using more than 22 devices. Considering this, the case was first divided into two types: errors in communication and errors in the sensor itself. Communication errors are divided into two types—periodic and aperiodic—considering the period. In the case of sensor error, a case was added assuming a rapid change in data and when the measurement range of the sensor itself was exceeded. We aimed to discover and suggest an appropriate imputation method, according to these four situations.

In cases of missing values, the univariate imputation and multivariate imputation techniques were applied, respectively. In univariate imputation, the existing univariate imputation technique was applied as is. For univariate imputation, linear interpolation (LI), spline interpolation (SI), last observation carried forward (LOCF), Kalman, and moving average (MA) methods were used. In multivariate imputation, five machine learning techniques were used, as follows: K-nearest neighbor (KNN), random forest (RF), linear regression (Reg), support vector machine (SVM), and miss forest (MF).

In order to consider both the time dependence of the univariate and the dependence of the multivariate on variables, an ensemble method was introduced, based on the predicted values from the univariate and multivariate. Among the ensemble methods, the weighted average and the stacking method were used. In the weighted average, different weights were set for univariate and multivariate data to obtain a weighted average. In addition, in the stacking, based on the values predicted by each model, the final meta-learner model was predicted to replace the missing values, again. 

The rest of the paper is organized as follows: Section 2 describes the experimental environment, existing imputation model, proposed ensemble method, and evaluation method. In Section 3, the authors aim to check the differences between models, according to the evaluation method, and compare the models that were finally used. In Section 4, the problem that occurred during the experiment and the points that were supplemented, confirmed through the results, are described. The final section addresses the paper’s conclusion.

## 2. Methodology

### 2.1. Experimental Setup and Dataset

**Figure 1:**
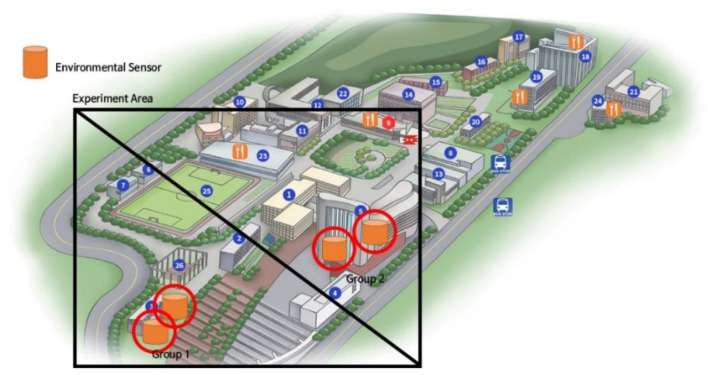
Location of the experiment sites in Soongsil University, divided into two groups: Group 1 and Group 2.

We formed an experimental group in Soongsil University to measure environmental sensors, as shown in Figure 1. This is because the indoor environment changes depending on the measurement location. When measuring multiple places, there is a disadvantage, in that the measuring range becomes excessively wide if it is measured from too far away [22]. Therefore, the environmental devices were placed in a line that could be controlled to some extent. Then, we checked that the linearity of the sensor was maintained, even if these two environments were slightly different. In Groups 1 and 2, places where people come in and out and places where people do not enter were set to reflect the effects on ventilation and movement.

**Figure 2:**
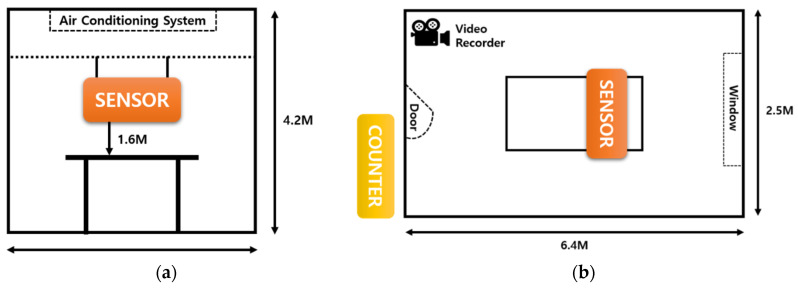
Experiment environment: (**a**) elevation view and (**b**) aerial view.

A total of 22 environmental sensor devices were used in the experiment. Twelve devices were set in Group 1, and 10 devices were set in Group 2. Group 1 was on the 5th floor and had a well-ventilated environment, while Group 2 was on the 1st basement floor and had a humid and low-temperature environment. For the devices used in the experiment that imputed missing values, 2 devices out of 12 were selected in Group 1. Two devices with linearity were selected: one device set for reference and the other device directly put into the situation of missing values. Experimental settings were created, as shown in Figure 2. The room size was 16 m^2^ and had an air conditioning system on the ceiling. This room was a meeting room, where people come and go. The obtained data were transmitted to the server using long-range (LoRa) communication, and the transmitted data were used for analysis.

**Figure 3:**
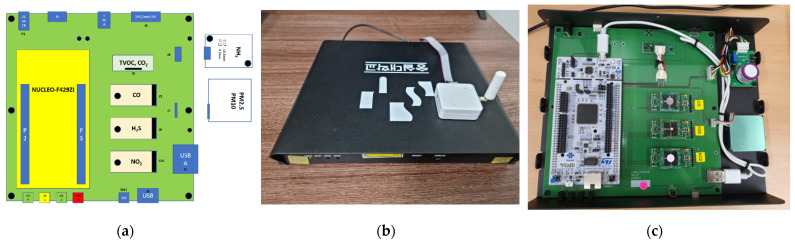
IoT environmental sensor device: (**a**) sensor device structure, (**b**) sensor device configuration, and (**c**) actual circuit diagram.

Our dataset contains environmental data collected from 10 gas sensors deployed in Soongsil University. As shown in Figure 3, the sensor device was equipped with a total of 10 environmental sensor modules, including those for temperature, humidity, CO, CO_2_, TVOC, PM2.5, PM10, NO_2_, NH_3_, and H_2_S. As a communication method, STM32F429ZIT MCU was used as a LoRa environmental sensor to collect information through a universal asynchronous receiver transmitter (UART), an inter-integrated circuit (I^2^C), and an analog–digital converter (ADC) for various environmental sensors, and an external LoRa modem, which also communicated using a UART. The control unit used a remote calibration protocol and performed functions such as resetting the device and changing the cycle of the sensor.

The data-collection period was measured from October 2020 to September 2021, and the data used for the experiment were from 5 March 2021 to 5 April 2021. Regarding the data interval, it was possible to secure about 16,000 pieces of data in 10 min intervals. Using real time series data makes more sense than using simulation data. There is a clear difference between real and simulated data [23]. This is because the data formation using simulation data and the technique used to fill it can lead to a different result when empirical data are received. The outline of specifications, according to the sensor type, are shown in Table 1.

### 2.2. Missing Data Imputation Methodology

**Figure 4:**
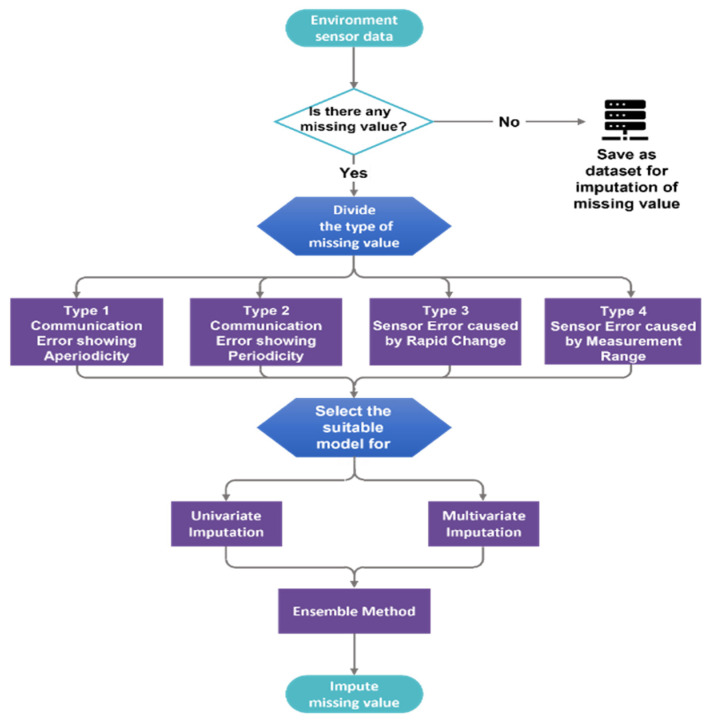
Flow chart of the process of imputing the missing value.

In many papers, when dealing with missing values, a dataset is obtained first, and then the missing values are generated. Missing values are randomly generated based on the missing completely at random (MCAR) process; however, this paper differs from previous papers, in that it considers the types of missing values separately and uses univariate and multivariate imputation at the same time, as shown in Figure 4.

In this experiment, the missing data ratio was set to several levels [24,25]. In this case, the reason for setting the missing data ratio differently was that each ratio had a different degree of influence on the data. When the missing rate was less than 1%, the effect was known to be negligible [26]. In addition, when the missing rate was between 1% and 5%, the data corresponded to manageable or flexible sample data. From the moment the missing rate reached 5% or more, a suitable solution was needed to handle missing values in the data [26]. From a missing rate of 15% or more, the missing value clearly affected the predictive model [27,28]. After generating missing values, various techniques were used to process them. Univariate imputation and multivariate imputation methods are usually used, depending on the data type. In univariate imputation, mean, mode, LI, SI, LOCF, Kalman, and MA methods are traditionally used. In multivariate imputation, KNN [29], RF [30], regression [31], SVM, and SVD [32] are traditionally used. Finally, using the above techniques, the missing data are usually processed by evaluation (MAE, RMSE, etc.) through a comparison between the predicted missing value and the actual value [33,34]. Since the data collected by the smart environmental sensors were time series data, which were sequentially collected, and various environmental factors must be considered together, we propose an algorithm that uses both methods together. The predictions from each imputation were collected and the weighted average and stacking algorithms were used to lower the evaluation values of the missing values.

### 2.3. Missing Data Type

The types of missing values are usually classified into three mechanisms, defined by Little and Rubin in 1987. These mechanisms are missing completely at random (MCAR), missing at random (MAR), and not missing at random (NMAR) [9]. In addition to the typical missing value types, defined by Little and Rubin, missing value cases were defined considering the characteristics of each sensor type, identified in Section 2.1. As can be seen in Table 2, we first divided the missing values into two cases: communication error and sensor error. In the communication error cases, the types of missing value were divided into two types: aperiodic and periodic. In the sensor error cases, the missing types were divided into rapid change and measurement range.

#### 2.3.1. Communication Error Cases

**Figure 5:**
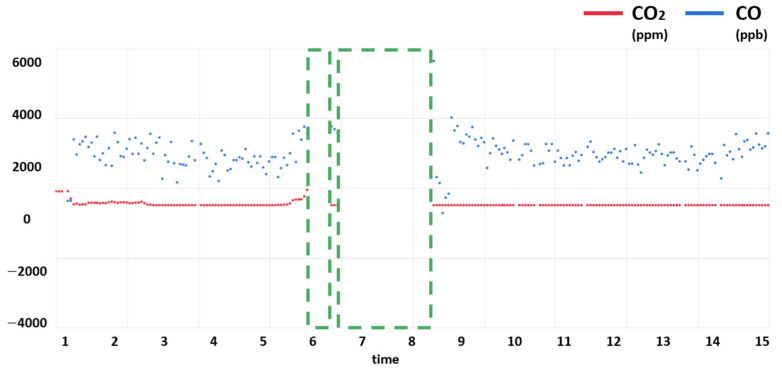
Missing values occur in real device using LoRa communication methods.

Communication instability was the most common case of missing values. This is the unavoidable task of IoT sensors operating in a wireless environment. As the device used in our experiment also used a communication method called long-range communication (LoRa), many errors were made in the communication terminal. LoRa has the advantages of having low power and a wide range, but LoRa with a low-power, wide-area network (LPWAN) has the disadvantage of a low transmission rate. In the experiment measuring the controlling switch in Nur-A-Alam, a signal loss of 9% was produced [35]. In Basford’s experiment, over 20 devices sent 135,000 messages, but only 72.4% were received by the server [36]. By checking the transmission rate using the received signal strength indicator (RSSI) in our sensor, it was confirmed that a similar problem occurred. A missing value for communication errors occurred in the LoRa-based environmental sensor device in use, as shown in Figure 5. As can be seen from Figure 5, the missing values occurred in two periods, the first lasting about an hour and the second communication error lasting about 6 h. As such, defects in communication in actual sensors usually occur sporadically, and one communication error often causes explosive communication errors.

Aperiodic and periodic missing values were classified within the communication errors category. It is common for missing values to occur completely randomly over time. However, since missing values may appear periodically, due to any cause, it was considered meaningful to devise a method to handle missing values in such cases. The periodic signal was generated according to the missing rate. The initial missing points were randomized and periodically generated.

**Figure 6:**
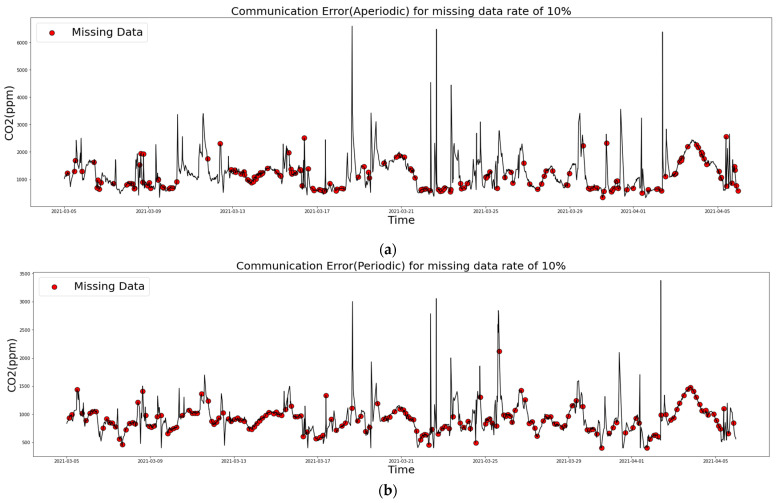
Missing values of CO_2_ with missing rate 10%: (**a**) communication error (aperiodic) and (**b**) communication error (periodic).

The graph shown in Figure 6 is the result of introducing missing values to the communication error case. This was the situation for CO_2_, and the missing rate was 10%. Figure 6a,b correspond to communication errors—periodic and aperiodic errors, respectively—therefore, as shown in (b), it can be confirmed that missing values occurred at regular intervals.

#### 2.3.2. Sensor Error Cases

The error types that occurred in the sensor itself are classified. First, missing values occurred when the measured values of the sensor changed rapidly. This often occurs in special circumstances, such as when someone smokes a cigarette. In consideration of situations in which the sensor could not detect a sudden change in data, a missing value was generated when the slope between the data was greater than or equal to a certain value. 

**Figure 7:**
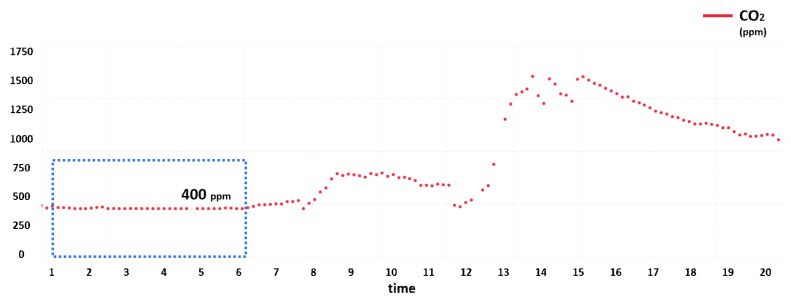
SVM 30 CO_2_ sensor data.

In addition, missing values can occur according to the measurement range of the sensor. The most affected factor was CO_2_, and a problem could be found, where the range of the CO_2_ sensor usually starts at 400 ppm. Since the lowest CO_2_ concentration in the atmosphere is 400 ppm, values cannot be measured below 400 ppm. The detection range of SVM 30—the CO_2_ sensor of the device we used—started at 400 ppm, and the same was true of UA50-VOC, which was a separate measurement module. Figure 7 shows the SVM 30 module measurements from 2 September 2021, and it can be seen that the CO_2_ value between 02:00 and 08:00 was fixed at 400 ppm.

**Figure 8:**
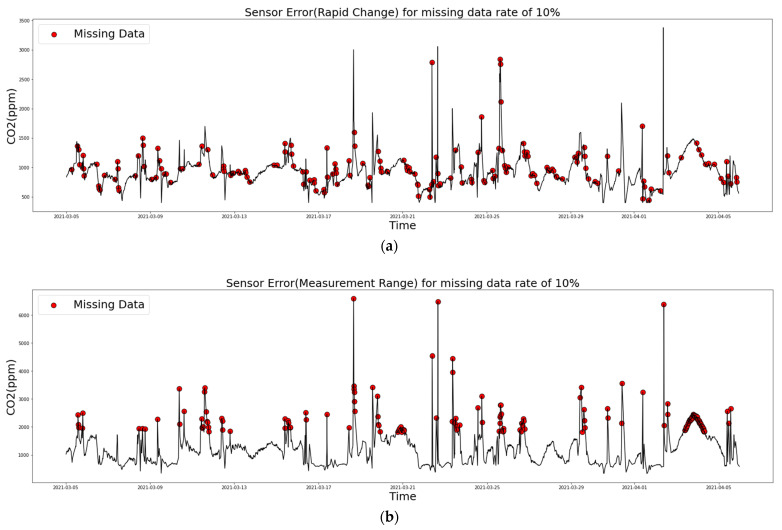
Missing values of CO_2_ with missing rate 10%: (**a**) Sensor Error (rapid change) and (**b**) Sensor Error (measurement range).

In Figure 8a,b show when an error occurred in the sensor. This was the same situation as seen above for CO_2,_ among other environmental substances, and the missing rate was 10%. Figure 8a shows a case where a missing value occurred when a rapid change occurred in the sensor, and Figure 8b shows a graph indicating when a certain measurement range in a sensor was exceeded.

The reason for dividing the cases like this is clear. First, it can be used as a background to select an appropriate imputation algorithm. Second, this knowledge helps to build a reasonable simulator that can eliminate missing values [18]. In addition to these cases, there were many cases where missing values occurred, but the four most frequent cases were selected. It is also necessary to consider additional situations, such as the occurrence of missing values due to human error or power supply.

### 2.4. Missing Value Imputation by Single Model

The method of imputation was divided into univariate imputation using time dependence, and multivariate imputation using the correlation between variables. In addition, the methods that were mainly used in each method are the traditional methods, because the existing models are reliable, fast, and uncomplicated [37]. 

#### 2.4.1. Imputation in Univariate Data

**Figure 9:**
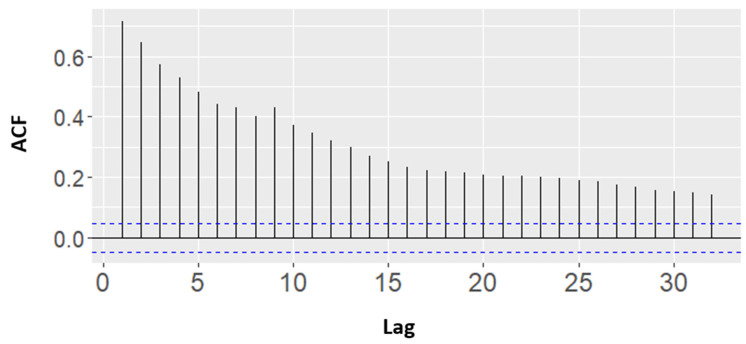
Autocorrelation coefficient for CO_2_.

Univariate time series data form a sequence of single observations at successive timepoints. Although usually considered a column of observations, time is actually an implicit variable [18]. The methods used in this section were replacement methods using time dependency. Therefore, as shown in Figure 9, the value of the autocorrelation function (ACF) exceeded the upper limit, so there was an autocorrelation.

The method of univariate imputation used here was as follows:

1. Linear interpolation: To estimate the missing value, the value of both endpoints was used to linearly estimate the missing value, according to the linear distance. LI was used to improve missing value replacement performance in the field of genotype replacement and machine translation [38,39].
2. Spline interpolation: Estimate missing values, using low-order polynomials, by dividing them into subintervals. This is also used to replace solar data and is being developed as a method for a distributed data modeling called thin-plate spline interpolation [40,41].
3. Last observation carried forward imputation (LOCF): Estimate missing values using data gathered just before the occurrence of missing values. This method is often used in longitudinal studies.
4. Moving average imputation: Estimate the missing value as the average of a window of a certain size around the missing value. This technique is mainly used for time series data.
5. Kalman imputation: Estimate missing values using Kalman smoothing. There was also a recent study on the treatment of missing values for local climate information [42].

#### 2.4.2. Imputation in Multivariate Data

Multivariate data are data with multiple independent variables. The methods used in this part were substitution methods, using dependencies between variables. Therefore, in our experimental data, we first examined the correlation between the variables using the Pearson correlation coefficient. 

**Figure 10:**
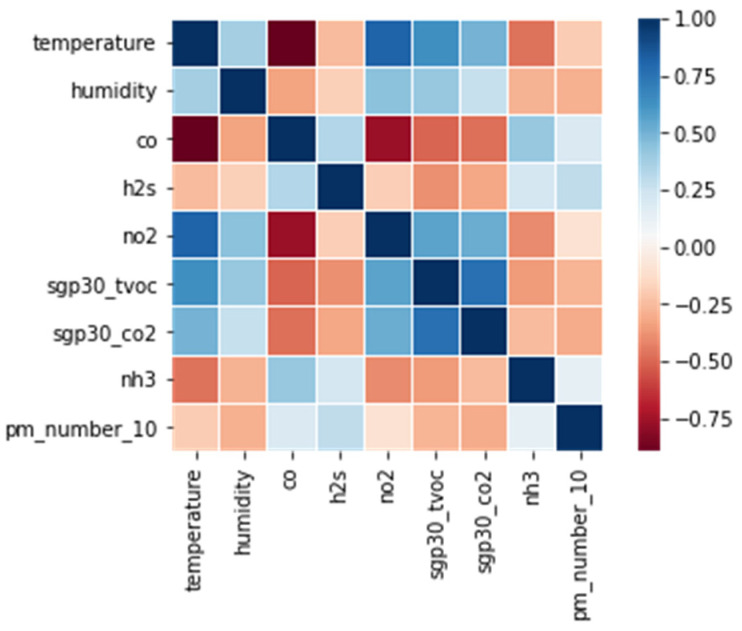
Pearson Correlation between environmental substances.

As shown in Figure 10, it is possible to identify environmental substances with strong correlations. For example, in environmental sensor data, there are high correlations, such as CO-temperature, NO_2_-temperature, CO_2_-TVOC, and NO_2_-CO. Before multivariate imputation was performed for each variable, feature selection was performed with variables showing a high correlation. This was because imputation with variables with clear correlations would be more effective than including all 10 variables in multivariate imputation.

The method of multivariate imputation used in this case was as follows:

1. K-NN imputation: The imputation of missing values using the k-values closest to the missing values. Based on this technique, a number of new, modified missing value imputation methods are emerging [43].
2. Multiple linear regression: Fitting a multiple linear regression model and replacing missing values using this. This is used in the imputation method of missing values to measure pollution concentration and air quality [44].
3. Random forest regression: Replacing missing values using the average predictions of multiple decision trees. Similar to K-NN, there are many new, modified missing value imputation methods based on random forest regression [45].
4. Support vector regression: A method using a support vector machine, which is used to replace missing values.
5. Miss forest: This is a random forest-based model, which is used to replace missing values. It can be used universally, regardless of continuous, categorical, or complex interactions and non-linear relationships [46].

### 2.5. Ensemble Learning Method

In this paper, we propose a statistical technique and a machine learning technique, respectively, as ensemble methods to consider the univariate imputation and multivariate imputation methods at the same time. A weighted average method that is easy to use and has a fast calculation speed was used as a statistical technique. A stacking method that predicts the final result, by building a prediction model using the result predicted by each substitution method, is used as a machine learning technique. 

#### 2.5.1. Weighted Average Method

The weighted average is one of the simple combination methods used in the ensemble method [47]. A weighted average was set by setting weights, and a proposal was made as a final result. High weights were given to methods with good performances, and low weights were set for methods with relatively poor performances. Weights were set in inverse proportion to the evaluation methods (MAE, RMSE) obtained from each imputation method. Equation (1) shows the result ($\hat{y}$) that was obtained after introducing the weighted average algorithm.

(1) $$\hat{y}=\frac{e_{2}}{e_{1}+e_{2}}{\hat{y}}_{1}+\frac{e_{1}}{e_{1}+e_{2}}{\hat{y}}_{2}\;$$

In this case, $\hat{y}$ is the final result prediction vector, and ${\hat{y}}_{1}$ and ${\hat{y}}_{2}$ are the predicted result vectors in univariate imputation and multivariate imputation. $e_{1}$ is the evaluation result value derived from univariate imputation and $e_{2}$ indicates the evaluation result value, derived from multivariate imputation.

#### 2.5.2. Stacking Method

**Figure 11:**
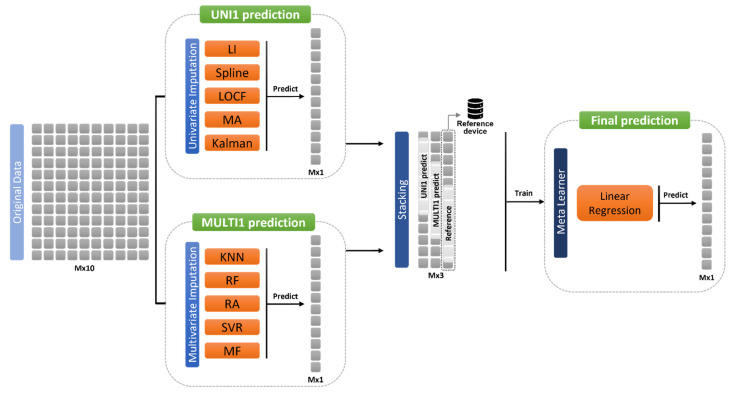
Diagram of stacking method using univariate and multivariate imputations for base learner.

The stacking method is a machine learning technique of the ensemble techniques used, along with bagging and boosting techniques, to make another prediction based on the data predicted by individual algorithms. This model considers the predictions of the base learner as new data, and trains them as a meta-learner, which helps to obtain more accurate predictions of the dataset [48]. A variety of base learner models can be applied to form a stacking model, and we chose the univariate imputation technique and the multivariate imputation technique for the base learner model. The basic performance of our stacking algorithm can be seen in Figure 11.

As can be seen in Figure 11, the original data for 10 types of environmental substances entered the base learner’s input variable. The base-learner consisted of the models used in Section 2.4.1 and Section 2.4.2. Thereafter, one of the five techniques of univariate imputation was selected to collect the missing value replacement values predicted by this technique. Multivariate imputation similarly collects the substitution value of one of the five techniques. When collecting environmental sensor data, data on the reference device was also collected, which was used as label data in the process of training the meta-learner. In conclusion, the substitution value for each technique in univariate and multivariate imputation and the sensor value in the reference device were integrated to enter the input variable (Mx3) of the meta-learner. In this case, the size of the input variable varied according to the missing value ratio. Then, the meta-learner model and the linear regression model were selected to predict the imputation value of the missing values. The predicted value was finally compared with the original data.

Algorithm 1 was followed as the stacking algorithm. In this algorithm, D_1_ and D_2_ are univariate and multivariate data, respectively. First, D_1_ and D_2_ are trained using U, which is a univariate imputer model used as a base learner, and M, which is a multivariate imputer model. Re-training is performed using the stacking imputer S, using P_1_ and P_2_, which are the predicted values of the learned data. In this case, R is used, which is a label in the reference device. In conclusion, the final predicted value, P_3_, is obtained.
**Algorithm 1.** Stacking Method.1: Step 1-1: univariate imputation 2: $D_{1\left(m\times 1\right)}={\left\{y_{i}\right\}}_{i=1}^{m}$: univariate missing data, *U:* univariate imputer model 3: $P_{1\left(m\times 1\right)}={\left\{{\hat{y}}_{i1}\right\}}_{i=1}^{m}$: imputed by *U* 4: Step 1-2: multivariate imputation 5: $T_{\left(n\times \left(p+1\right)\right)}={\left\{y_{i},x_{i1},x_{i2},\cdots ,x_{ip}\right\}}_{i=1}^{n}$: multivariate data (no missing), $D_{2\left(m\times p\right)}={\left\{x_{i1},x_{i2},\cdots ,\;x_{ip}\right\}}_{i=1}^{m}$: multivariate missing data, *M:* multivariate imputer model 6: M train by *T* 7: $P_{2\left(m\times 1\right)}={\left\{{\hat{y}}_{i2}\right\}}_{i=1}^{m}$: imputed by (*M*,$\;D_{2}$) 8: Step 2: stacking method 9: $S_{d\left(m\times 2\right)}={\left\{{\hat{y}}_{i1},{\hat{y}}_{i2}\right\}}_{i=1}^{m}$: stack $P_{1}$ and $P_{2}$, *S:* stacking imputer, $R_{\left(m\times 1\right)}={\left\{Y_{i}\right\}}_{i=1}^{m}$: reference data 10: S train by (*R*, *S_d_*) 11: $P_{3\left(m\times 1\right)}={\left\{{\hat{y}}_{i}\right\}}_{i=1}^{m}$: imputed by *S* 12: $P_{3}$: final prediction values

### 2.6. Evaluation Method

To prove the effect of missing data imputation when applied to environmental sensor data, the evaluation method was measured with the mean absolute error (MAE) and the root mean square error (RMSE). MAE and RMSE are the most widely used evaluation methods for the imputation of missing values [49,50,51,52]. The formulas for these methods are shown in Table 3.

In this case, $x_{i}$ is the actual value of the environmental sensor data, ${\hat{x}}_{i}$ is the imputed value of the environmental sensor data, and n is the number of samples. When using RMSE, missing values are not biased and are used when the distribution is normal. On the other hand, MAE is suitable for evaluating uniformly distributed missing values [24]. Unlike MAE, RMSE gives a large penalty for values with a large error. These two methods performed different evaluations according to the distribution of and errors in the data. In this experiment, four cases of missing values were set. At this time, missing values were distributed differently for each case. Therefore, the distribution of errors was also expected to be different for each case. Therefore, by checking the MAE and RMSE at the same time, we could compare the performance regardless of the distribution of various errors by case.

## 3. Results

When using the ensemble model, a total of 25 cases were confirmed by introducing one technique each from 5 univariate techniques and 5 multivariate techniques. Among them, when comparing the existing model and the proposed model, the model with the best performance in the existing model was selected and compared.

### 3.1. Differences between Models According to Evaluation Method

**Figure 12:**
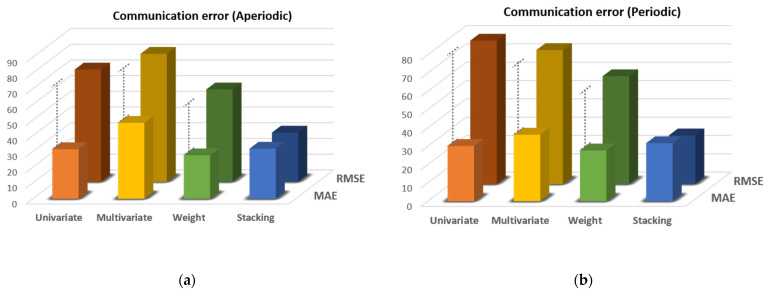

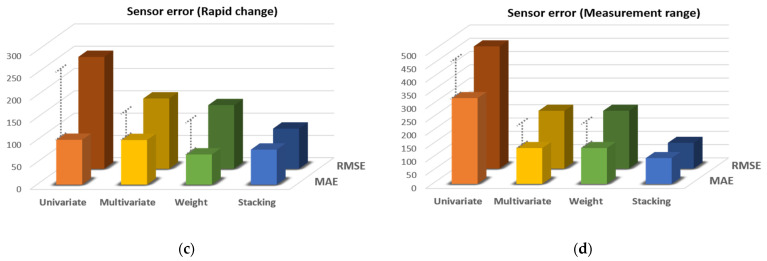
Comparison of MAE and RMSE values by imputation method for CO_2_ with missing rate 15%: (**a**) communication error (aperiodic), (**b**) communication error (periodic), (**c**) sensor error (rapid change), and (**d**) sensor error (measurement range).

Among the 10 environmental substances measured by the environmental sensor device, CO_2_ was mainly used in the results. Other environmental substances showed similar results, and CO_2_, which showed the clearest result, was selected. As mentioned in Section 2.6, the RMSE evaluation method showed a greater penalty for errors that deviated significantly from the MAE method. Through this, we tried to judge the characteristics of the model considering both MAE and RMSE. If the RMSE value was higher than the MAE value, this suggested that a large error has occurred for a specific missing value. Figure 12 is a graph showing the MAE and RMSE values of each technique, with a missing rate of 15%, and each of the four CO_2_ situations. As shown in Figure 12, univariate imputation, multivariate imputation, and weighted average methods show that the RMSE value tended to rise compared with the MAE value. On the other hand, the stacking method does not show a tendency to increase the RMSE value compared with the MAE value. It can be seen that stacking does not cause a large error. Looking at Figure 12a, when un ivariate imputation was applied, the MAE value was 31.59 and the RMSE was measured to be 71.51. When multivariate imputation was applied, the MAE was 48.36 and RMSE was 81.46, and when a weighted average was used, MAE was 27.70 and RMSE was 58.78. On the other hand, when stacking was used, the MAE was 31.92 and RMSE was 31.31; therefore, it can be confirmed that RMSE derives similar values to MAE, unlike the above three methods.

**Figure 13:**
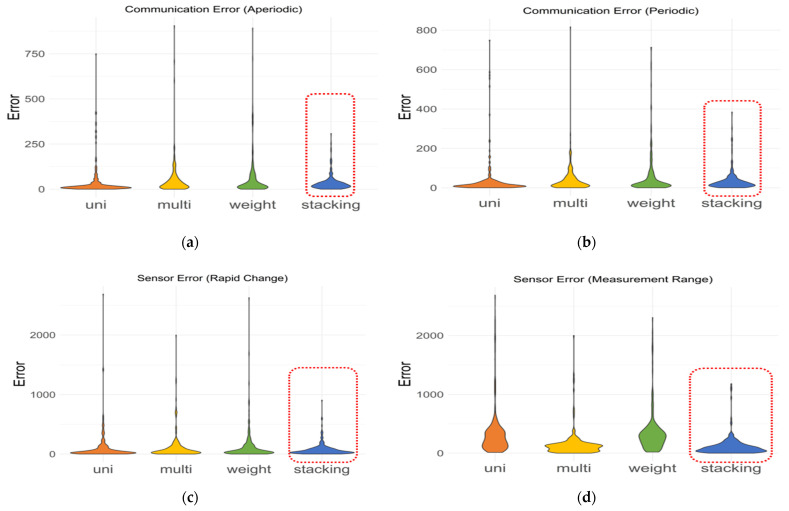
Comparison of error distribution values by imputation method for CO_2_ with missing rate 15%: (**a**) communication error (aperiodic), (**b**) communication error (periodic), (**c**) sensor error (rapid change), and (**d**) sensor error (measurement range).

The error distribution for the four cases can be checked in Figure 13. There was a large error in models, except for the stacking, and the distribution of errors in stacking was more stable than in other models. This means that the stacking method showed no significant deviations from the existing value, and it can be expected that the RMSE of the stacking method will not soon increase with respect to MAE. This can be confirmed from the stacking distribution of (a), (b), (c), and (d) of Figure 13.

### 3.2. Performance Comparison between Models

**Figure 14:**
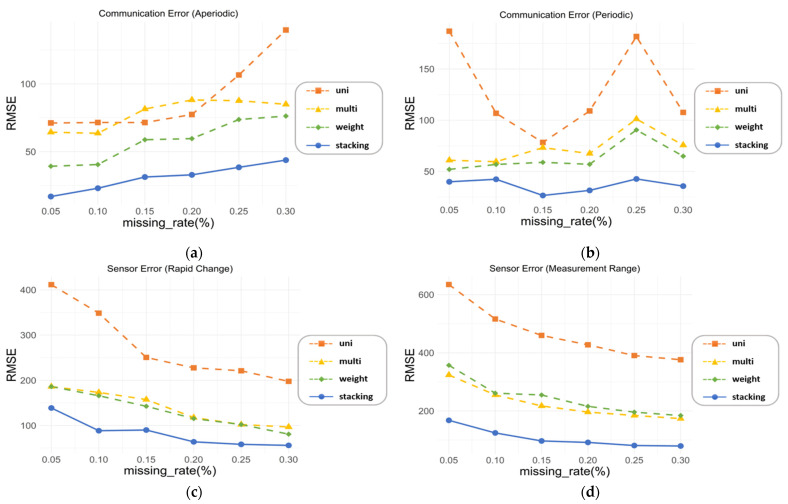
Comparison of RMSE by imputation method for CO_2_ with missing rates 5, 10, 15, 20, 25 and 30%: (**a**) communication error (aperiodic), (**b**) communication error (periodic), (**c**) sensor error (rapid change), and (**d**) sensor error (measurement range).

First, the target variable was set as CO_2_ from 10 types of environmental substances, and RMSE was set as the evaluation method. We aimed to compare the performance of different models according to the occurrence of missing values. In addition, the model’s performance was checked by varying the missing rate in to see the numerical values that affected the model, according to the missing rate. Assuming that the missing rates were 5, 10, 15, 20, 25, and 30%, we checked whether our ensemble method was suitable for use in diverse missing situations. As can be seen in Figure 14, it was confirmed that the missing rate in the four cases did not significantly affect the performance between models. In other words, it can be seen that the ensemble model performs better than the univariate imputation model and the multivariate imputation model, which are existing models, even if the missing rate changes. Looking at (a), (b), (c), and (d) in Figure 14, stacking performed the best regardless of the missing value case. The model using the weighted average performed better than the conventional method in Figure 14a, but slightly better than the multivariate imputation model in Figure 14b,c, and slightly worse than the multivariate imputation model in Figure 14d.

**Figure 15:**
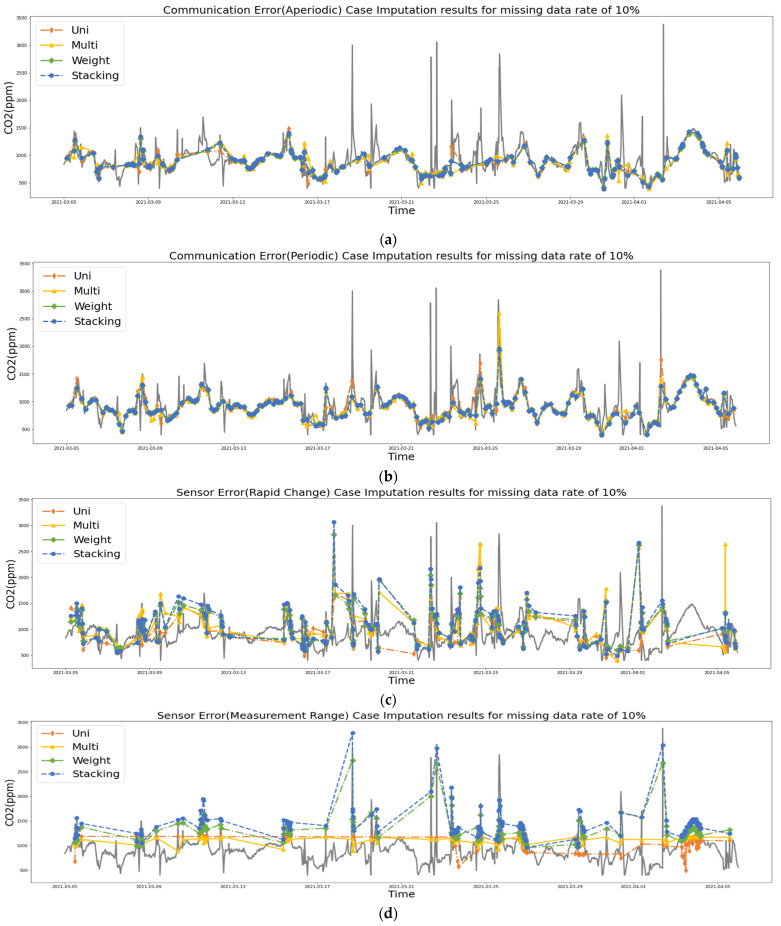
Imputation of missing values in the existing graph, according to the imputation method for CO_2_ with missing rate 10%: (**a**) communication error (aperiodic), (**b**) communication error (periodic), (**c**) sensor error (rapid change), and (**d**) sensor error (measurement range).

Figure 15 shows the imputation figure for the occurrence of four cases. Figure 15 shows the 10% missing rate for CO_2_, and shows a graph connected by a dotted line, based on the missing values imputed by each technique. In the sensor error cases shown in Figure 15c,d, it is easier to see that the weighted average and stacking imputation follow the existing graph well. As shown in Figure 15c, it can be seen that univariate and multivariate imputation replaced the outliers from the existing graph, while weighted average and stacking follow the existing graph. In particular, in Figure 15d, it can be seen that the stacking technique learns using the numerical values of the reference device, so it can be seen that the missing values are better predicted for the existing data.

Table 4 shows the RMSE evaluation result for CO_2_, and the missing rate was set as 10%. The final prediction, derived from a weighted average chosen from the ensemble methods, was better than or similar to the two methods of univariate and multivariate imputation, and it was confirmed that the performance was somewhat lower in the measurement range case compared with the sensor errors. On the other hand, when the stacking method was chosen from the ensemble methods, it can be seen that, in all four cases, the RMSE performance was better than the rest of the models.

Table 5 shows the model execution time for CO_2_, and the missing rate was set to 10%. The execution time of the existing model is the average execution time for 5 techniques, and the proposed model is the average execution time for 25 combinations. There seems to be no significant difference in execution time for each case. Comparing the existing model with the proposed model, since the proposed model performs additional work after executing two existing models, a longer execution time is required compared with the existing model. However, the complexity of the model does not seem to be a big problem as the time does not differ significantly compared to the existing model. In the proposed model, the time in parentheses means the time it takes to do additional work.

## 4. Discussion

When a missing value occurred in the environmental sensor, an ensemble imputation method was conducted according to the appropriate case. As mentioned in Section 2.3, we assumed the existence of four cases. This was derived from last year’s environmental sensor data measurement, and the four most frequently occurring cases were selected. In addition to this, several cases can be added for cases where missing values occur. Examples include limitations in data collection and human error in the storage process [5,10]. In this technique, errors in communication were divided only into errors in period. However, not only periodicity, but also various errors, were detected for communication error situations. For example, if one sensor causes a communication error on a device, other sensors are affected, or once a communication error occurs, successive transmission failure leads to a burst of losses. In order to develop such a more advanced technique, it is necessary to add and subdivide cases that actually occur for communication errors.

In the sensor error (measurement range) case of Table 4, the RMSE performance of the weighted average tended to be poorer than that of the other three cases. In the measurement range case, since a certain sensor range is set and values that surpass this were judged as missing, both univariate and multivariate imputation models tended to underpredict compared with the original missing value. However, since the weighted average was an ensemble technique that averaged the univariate and multivariate models by weighting them without a separate training process, it was difficult to derive a value close to the actual value. Therefore, as shown in Section 3.2, it can be seen that the weighted average model performed poorly for multivariate imputation in the measurement range case.

When retraining with the meta-learner, while performing the stacking method, we also considered which value should be set as a label. Unlike this study, if missing values occur in real devices, there is no label value. Therefore, there is a problem in training the stacking model at this time. We solved this problem through two devices, whose linearity was confirmed when setting the sensor. The time series data of the corresponding variable were obtained from a device with no missing values, and the ensemble method was introduced in the device with missing values. If a sufficient number of missing sample values are learned in the device setting process, it is expected that missing values will be properly replaced, even when missing values actually occur. This also solves the universal problem of not being able to evaluate the replacement technique when applied to a real device.

When operating an actual sensor, it is also necessary to consider whether the proposed technique will be effective even in dynamically changing situations. In the actual environment, unexpected problems occur, such as continuous missing values for a certain period of time, as shown in Figure 5. In order to introduce this technique in actual sensors, such cases should be further subdivided and added to further strengthen the natural induction of the correct replacement technique. In addition, if data is accumulated and learned for sufficient time in a situation where missing is minimized, missing values can be replaced well, even in situations that become dynamic in the future.

The ensemble method involves the application of the model based on the predicted or evaluated values of the existing univariate and multivariate imputation models. Therefore, we have no choice but to rely on the performance of univariate and multivariate imputation, which means that the performance of a single model should support the method. In other words, in order to increase the performance of the stacking algorithm, it is necessary to improve the performance of univariate and multivariate imputation first. This problem can be solved by boosting performance in our systematic confrontation process using the latest high-performance techniques, rather than universal techniques. 

## 5. Conclusions

Interest in the environment is growing and the reliability of environmental sensors that can measure it has been emphasized. In the process of collecting sensor data, some data may be lost, and it is important to deal with these missing values accordingly. Various methods of handling missing values are being studied, but a new method is needed for the more accurate replacement of missing values that can be applied to environmental sensors. In the experiment, a new ensemble method that considers time dependence and correlation with other environmental substances was proposed.

In this study, we first created four cases in which missing values can occur in environmental sensors. For each situation, five traditional univariate imputation techniques and five multivariate imputation techniques were applied. Then, weighted average and stacking models were applied to the ensemble methods, based on the missing values were predicted by each model. After that, we checked the difference between the actual value and our predicted missing value, shown through MAE and RMSE. In this process, the missing rates (5, 10, 15, 20, 25, and 30%) were changed to determine whether our ensemble method was effective in various situations. The experiment was conducted based on CO_2_, chosen from 10 environmental substances. As shown in Section 3.2, when the missing rate is 10%, it could be seen that the stacking performance of the ensemble method was measured more accurately than the other three models. It showed a good performance in all four cases. In addition to this, it was confirmed that the stacking method had the best performance among the ensemble methods, and the weighted average showed a good performance, even when the missing rate was changed. As well as CO_2_, the ensemble method was used for 10 types of sensor data to determine whether a good performance could be derived for other environmental materials.

The most significant element to emphasize concerning the proposed technique is its usability. This technique can be applied to all sensors using multivariate among time series data. We tried to implement a lightweight, yet easy-to-implement, technique by using the most common techniques in replacing missing values as base learners of ensemble techniques. In addition, the base learner does not influence which technique is included; therefore, it is a simplified algorithm that does not have a problem using the latest technique for the base learner.

In addition, existing papers have not divided the situation in which missing values occur when implementing an algorithm for missing value replacement. Usually, the existing papers were conducted only by changing the missing rate. However, we have divided the situation in which missing values occur into four cases and established a countermeasure against errors that actually occur frequently. These cases can be added at any time, and by establishing a countermeasure against these cases, there is a process of recommending and introducing appropriate confrontation techniques when missing occurs.

The imputation of such missing values is required not only in environmental sensors, but also in various fields such as the smart city. When missing values occur in the environmental sensor, our new ensemble method that considers the time dependence and the correlation between variables can be significantly contributed.

## Figures and Tables

**Table 1:** Measurements of performance characteristics of the sensor used on the device.

Sensor Model	Sensor Type	Sensing Target	Detection Range
SPS 30	Optical	PM1, PM2.5, PM4, PM10	1–1000 μg/m^3^
SVM 30	Semiconductor	TVOC, CO_2_, Temperature, Humidity	TVOC: 0~60 ppm, CO_2_: 0~60,000 ppm, Temperature: –20~85 °C, Humidity: 0~100% RH
DGS-CO 968-034	Electrochemical	CO	0–1000 ppm
DGS-H_2_S 968-036		H_2_S	0–10 ppm
DGS-NO_2_ 968-043		NO_2_	0–5 ppm
FECS44-100		NH_3_	0–100 ppm

**Table 2:** Classified occurrence case of missing values.

Case	Missing Type	Missingness Mechanism
Communication error	Aperiodic	MCAR
	Periodic	MCAR
Sensor error	Rapid change	NMAR
	Measurement Range	NMAR

**Table 3:** Evaluation method.

Evaluation Method	Equation	Perfect Score	Data Distribution
Mean Absolute Error (MAE)	MAE = $\frac{1}{n}\sum_{i=1}^{n}\left|x_{i}-{\hat{x}}_{i}\right|$	0	Uniform distribution
Root Mean Square Error (RMSE)	RMSE = $\sqrt{\frac{1}{n}\sum_{i=1}^{n}{\left|x_{i}-{\hat{x}}_{i}\right|}^{2}}$	0	Normal distribution

**Table 4:** Comparison of RMSE between existing model and proposed model, according to the missing value occurrence case for CO_2,_ with missing rate 10%.

Case	Existing Model		Proposed Model	
	Univariate	Multivariate	Weighted Average	Stacking
Communication error (aperiodic)	71.51	63.57	40.48	**23.00**
Communication error (periodic)	106.73	59.52	56.82	**42.33**
Sensor error (rapid change)	348.51	173.37	165.97	**88.53**
Sensor error (measurement range)	515.92	255.14	298.88	**124.31**

**Table 5:** Comparison of average execution time between existing model and proposed model, according to the missing value occurrence case for CO_2_, with missing rate 10%.

Case	Existing Model		Proposed Model	
	Univariate (A)	Multivariate (B)	Weighted Average (C) (C-A-B)	Stacking (D) (D-A-B)
Communication error (aperiodic)	0.009	0.814	0.847	0.836
			(0.024)	(0.013)
Communication error (periodic)	0.009	0.814	0.837	0.842
			(0.014)	(0.019)
Sensor error (rapid change)	0.009	0.808	0.840	0.831
			(0.023)	(0.014)
Sensor error (measurement range)	0.008	0.807	0.826	0.836
			(0.011)	(0.021)

## Data Availability

Not applicable.

## References

[1] Metia S., Ha Q., Duc H., Scorgie Y. (2020). Urban air pollution estimation using unscented Kalman filtered inverse modeling with scaled monitoring data. Sustain. Cities Soc..

[2] Cho J., Joo W. (2020). Data Clustering Method Using Efficient Fuzzifier Values Derivation. IEEE Access.

[3] Wang J., Dong K. (2019). What drives environmental degradation? Evidence from 14 Sub-Saharan African countries. Sci. Total Environ..

[4] WHO. https://www.who.int/vietnam/news/feature-stories/detail/ten-threats-to-global-health-in-2019.

[5] Xu X., Nie S., Ding H., Hou F.F. (2018). Environmental pollution and kidney diseases. Nat. Rev. Nephrol..

[6] Liang J., Qin Y., Hong Z. (2007). An Auto-exposure algorithm for detecting high contrast lighting conditions. Proceedings of the 2007 7th International Conference on ASIC.

[7] Liu Y., Dillon T., Yu W., Rahayu W., Mostafa F. (2020). Missing Value Imputation for Industrial IoT Sensor Data with Large Gaps. IEEE Internet Things J..

[8] Panapakidis I.P., Bouhouras A.S., Christoforidis G.C. (2018). A missing data treatment method for photovoltaic installations. Proceedings of the 2018 IEEE International Energy Conference (ENERGYCON).

[9] Little R.J., Rubin D.B. (2019). Statistical Analysis with Missing Data.

[10] Cismondi F., Fialho A.S., Vieira S.M., Reti S.R., Sousa J.M.C., Finkelstein S.N. (2013). Missing data in medical databases: Impute, delete or classify?. Artif. Intell. Med..

[11] Graham J.W. (2009). Missing Data Analysis: Making It Work in the Real World. Annu. Rev. Psychol..

[12] García-Laencina P.J., Sancho-Gómez J.-L., Figueiras A.R. (2009). Pattern classification with missing data: A review. Neural Comput. Appl..

[13] Sedghi S., Sadeghian A., Huang B. (2017). Mixture semisupervised probabilistic principal component regression model with missing inputs. Comput. Chem. Eng..

[14] Khatibisepehr S., Huang B. (2008). Dealing with Irregular Data in Soft Sensors: Bayesian Method and Comparative Study. Ind. Eng. Chem. Res..

[15] Magnani M. (2004). Techniques for Dealing with Missing Data in Knowledge Discovery Tasks. http://magnanim.web.cs.unibo.it/index.html.

[16] Huamin T., Qiuqun D., Shanzhu X. (2020). Reconstruction of time series with missing value using 2D representation-based denoising autoencoder. J. Syst. Eng. Electron..

[17] Bhandari S., Bergmann N., Jurdak R., Kusy B. (2017). Time Series Analysis for Spatial Node Selection in Environment Monitoring Sensor Networks. Sensors.

[18] Moritz S., Sardá A., Bartz-Beielstein T., Zaefferer M., Stork J. (2015). Comparison of different methods for univariate time series imputation in R. arXiv.

[19] Baddoo T., Li Z., Odai S., Boni K., Nooni I., Andam-Akorful S. (2021). Comparison of Missing Data Infilling Mechanisms for Recovering a Real-World Single Station Streamflow Observation. Int. J. Environ. Res. Public Health.

[20] Yan X., Xiong W., Hu L., Wang F., Zhao K. (2015). Missing Value Imputation Based on Gaussian Mixture Model for the Internet of Things. Math. Probl. Eng..

[21] Park J., Kim S. (2020). Improved Interpolation and Anomaly Detection for Personal PM2.5 Measurement. Appl. Sci..

[22] Chen L.-J., Ho Y.-H., Hsieh H.-H., Huang S.-T., Lee H.-C., Mahajan S. (2018). ADF: An Anomaly Detection Framework for Large-Scale PM2.5 Sensing Systems. IEEE Internet Things J..

[23] Apostol E.-S., Truică C.-O., Pop F., Esposito C. (2021). Change Point Enhanced Anomaly Detection for IoT Time Series Data. Water.

[24] Crespo Turrado C., Sánchez Lasheras F., Calvo-Rollé J.L., Piñón-Pazos A.J., de Cos Juez F.J. (2015). A New Missing Data Imputation Algorithm Applied to Electrical Data Loggers. Sensors.

[25] Kim T., Ko W., Kim J., Kim T. (2019). Analysis and Impact Evaluation of Missing Data Imputation in Day-ahead PV Generation Forecasting. Appl. Sci..

[26] Batista G., Monard M.C. (2003). An analysis of four missing data treatment methods for supervised learning. Appl. Artif. Intell..

[27] Banks D., House L., McMorris F.R., Arabie P., Gaul W.A. (2011). Classification, Clustering, and Data Mining Applications. Proceedings of the Meeting of the International Federation of Classification Societies (IFCS), Illinois Institute of Technology.

[28] Luengo J., García S., Herrera F. (2010). A study on the use of imputation methods for experimentation with radial basis function network classifiers handling missing attribute values: The good synergy between rbfns and eventcovering method. Neural Netw..

[29] Brock G.N., Shaffer J.R., E Blakesley R., Lotz M.J., Tseng G.C. (2008). Which missing value imputation method to use in expression profiles: A comparative study and two selection schemes. BMC Bioinform..

[30] Xia J., Zhang S., Cai G., Li L., Pan Q., Yan J., Ning G. (2017). Adjusted weight voting algorithm for random forests in handling missing values. Pattern Recognit..

[31] Burgette L.F., Reiter J.P. (2010). Multiple Imputation for Missing Data via Sequential Regression Trees. Am. J. Epidemiol..

[32] Kang P. (2013). Locally linear reconstruction based missing value imputation for supervised learning. Neurocomputing.

[33] Gautam C., Ravi V. (2015). Data imputation via evolutionary computation, clustering and a neural network. Neurocomputing.

[34] Silva-Ramírez E.-L., Pino-Mejías R., López-Coello M. (2015). Single imputation with multilayer perceptron and multiple imputation combining multilayer perceptron and k-nearest neighbours for monotone patterns. Appl. Soft Comput..

[35] Ahsan M., Based M., Haider J., Rodrigues E.M. (2021). Smart Monitoring and Controlling of Appliances Using LoRa Based IoT System. Designs.

[36] Basford P.J., Bulot F.M.J., Apetroaie-Cristea M., Cox S.J., Ossont S.J.J. (2020). LoRaWAN for Smart City IoT Deployments: A Long Term Evaluation. Sensors.

[37] Cho J. (2022). Efficient Autonomous Defense System Using Machine Learning on Edge Device. CMC-Computers.

[38] Browning B.L., Browning S. (2016). Genotype Imputation with Millions of Reference Samples. Am. J. Hum. Genet..

[39] Li Y., Li J., Zhang M., Li Y., Zou P. (2020). Improving Neural Machine Translation with Linear Interpolation of a Short-Path Unit. ACM Trans. Asian Low-Resour. Lang. Inf. Process..

[40] Karim S.A.A., Ismail M.T., Othman M., Abdullah M.F., Hasan M.K., Sulaiman J. (2018). Rational cubic spline interpolation for missing solar data imputation. J. Eng. Appl. Sci..

[41] Keller W., Borkowski A. (2019). Thin plate spline interpolation. J. Geod..

[42] Saputra M.D., Hadi A.F., Riski A., Anggraeni D. (2021). Handling Missing Values and Unusual Observations in Statistical Downscaling Using Kalman Filter. J. Phys. Conf. Ser..

[43] Huang J., Keung J., Sarro F., Li Y.-F., Yu Y., Chan W.K., Sun H. (2017). Cross-validation based K nearest neighbor imputation for software quality datasets: An empirical study. J. Syst. Softw..

[44] Shahbazi H., Karimi S., Hosseini V., Yazgi D., Torbatian S. (2018). A novel regression imputation framework for Tehran air pollution monitoring network using outputs from WRF and CAMx models. Atmos. Environ..

[45] Kokla M., Virtanen J., Kolehmainen M., Paananen J., Hanhineva K. (2019). Random forest-based imputation outperforms other methods for imputing LC-MS metabolomics data: A comparative study. BMC Bioinform..

[46] Stekhoven D.J., Bühlmann P. (2012). MissForest—non-parametric missing value imputation for mixed-type data. Bioinformatics.

[47] Li J., Yu Y., Qing X. (2021). Embedded FBG Sensor Based Impact Identification of CFRP Using Ensemble Learning. Sensors.

[48] Xu Y., Meng R., Zhao X. (2021). Research on a Gas Concentration Prediction Algorithm Based on Stacking. Sensors.

[49] Li L., Li Y., Li Z. (2013). Efficient missing data imputing for traffic flow by considering temporal and spatial dependence. Transp. Res. Part C Emerg. Technol..

[50] Smith B.L., Scherer W.T., Conklin J.H. (2003). Exploring Imputation Techniques for Missing Data in Transportation Management Systems. Transp. Res. Rec. J. Transp. Res. Board.

[51] Chen M., Xia J., Liu R.R. (2010). Developing a Strategy for Imputing Missing Traffic Volume Data. J. Transp. Res. Forum.

[52] Chai T., Draxler R.R. (2014). Root mean square error (RMSE) or mean absolute error (MAE)?—Arguments against avoiding RMSE in the literature. Geosci. Model Dev..

